# GIP_HUMAN[22–51] is a new proatherogenic peptide identified by native plasma peptidomics

**DOI:** 10.1038/s41598-021-93862-w

**Published:** 2021-07-14

**Authors:** Tsuguto Masaki, Yoshio Kodera, Michishige Terasaki, Kazumi Fujimoto, Tsutomu Hirano, Masayoshi Shichiri

**Affiliations:** 1 Department of Endocrinology, Diabetes and Metabolism, Kitasato University School of Medicine, 1-15-1 Kitasato, Minami-ku, Sagamihara, Kanagawa 252-0374 Japan; 2 Department of Physics, Center for Disease Proteomics, Kitasato University School of Science, 1-15-1 Kitasato, Minami-ku, Sagamihara, Kanagawa 252-0373 Japan; 3 Division of Diabetes, Metabolism and Endocrinology, Department of Medicine, Showa University School of Medicine, 1-5-8 Hatanodai, Shinagawa-ku, Tokyo, 142-8555 Japan; 4 Present Address: Tokyo Kyosai Hospital, 2-3-8 Nakameguro, Meguro-ku, Tokyo, 153-8934 Japan

**Keywords:** Atherosclerosis, Mass spectrometry, Peptides, Endocrinology

## Abstract

We recently established a new plasma peptidomic technique and comprehensively identified a large number of low-molecular weight and low-abundance native peptides using a single drop of human plasma. To discover a novel polypeptide that potently modulates the cardiovascular system, we performed a bioinformatics analysis of the large-scale identification results, sequentially synthesized the selected peptide sequences, tested their biological activities, and identified a 30-amino-acid proatherogenic peptide, GIP_HUMAN[22–51], as a potent proatherosclerotic peptide hormone. GIP_HUMAN[22–51] has a common precursor with the glucose-dependent insulinotropic polypeptide (GIP) and is located immediately N-terminal to GIP. Chronic infusion of GIP_HUMAN[22–51] into *ApoE*^*−/−*^ mice accelerated the development of aortic atherosclerotic lesions, which were inhibited by co-infusions with an anti-GIP_HUMAN[22–51] antibody. GIP_HUMAN[22–51] increased the serum concentrations of many inflammatory and proatherogenic proteins, whereas neutralising antibodies reduced their levels. GIP_HUMAN[22–51] induced IκB-α degradation and nuclear translocation of NF-κB in human vascular endothelial cells and macrophages. Immunoreactive GIP_HUMAN[22–51] was detected in human tissues but there was no colocalization with the GIP. The plasma GIP_HUMAN[22–51] concentration in healthy humans determined using a stable-isotope tagged peptide was approximately 0.6 nM. This study discovered a novel endogenous proatherogenic peptide by using a human plasma native peptidomic resource.

## Introduction

The glucose-dependent insulinotropic polypeptide (GIP) is a 42-amino-acid polypeptide secreted by enteroendocrine K-cells^1^ that potentiates the glucose-dependent release of insulin from pancreatic β cells^2,3^ and exerts extrapancreatic glucoregulatory activities through its systemic receptors^4^. GIP is encoded by the GIP gene and biosynthesized from the 153 amino acid precursor protein by removal of the flanking 30- and 81-amino-acid extensions at the N- and C-terminals, respectively^5^. The C-terminal end amino acid residue of the N-terminal peptide, arginine^6,7^, is thought to be removed by carboxypeptidases during peptide biosynthesis^6,8,9^. However, the presence and exact amino acid sequence of such endogenously processed peptides in human plasma remain largely unknown because of the technical difficulties associated with plasma peptidomic analysis. Furthermore, this putative N-terminal peptide has no known biological activity.

Although human plasma represents an informative resource describing the proteome^10^, identifying plasma bioactive peptides and disease biomarkers remains extremely difficult because these endogenous peptides exist at trace levels, while the plasma contains an extraordinarily dynamic range of high-molecular-weight proteins^11,12^. Thus, direct in-depth plasma peptidomic profiling remains challenging in contemporary analytical biochemistry^13,14^. We previously identified novel bioactive peptides by predicting putative endogenous peptide sequences from the bioinformatic analysis of human cDNA database information, explored biological activities of the synthesised peptides, and confirmed their immunoreactive presence in human plasma and tissues^15^. This method identified potent bioactive peptides with physicochemical characteristics that prevented their identification by conventional methods^16^. An in silico search for peptides with specific functional motifs was used to discover bioactive peptides^17–19^. However, to complete the discovery process of putative peptide hormones, their exact native amino acid sequences in the peripheral circulation needs to be confirmed by mass spectrometry^20^. Despite long-term endeavours to elucidate the plasma proteome^11,21^, the comprehensive identification of plasma low-molecular weight native peptides has not been achieved until very recently^22^.

In a recent study, we established improved technology that improved the high-yield plasma extraction technique^22^, enabling the large-scale identification of plasma native peptides with mass spectrometry. We deposited the results into a newly generated human plasma native peptidomic database. We also synthesised peptides of identified sequences and validated their biological activity using cultured human cells. The present study aimed to use this plasma native peptidomic resource to identify a novel plasma native polypeptide hormone that may be involved in the pathophysiology of vascular diseases.

## Results

### In silico analysis of the mass spectrometry-identified human plasma native peptidomic resource

Our initial peptidomic sequencing data acquired from 189 analyses using liquid chromatography tandem mass spectrometry (LC–MS/MS) were searched against the SwissProt_2015_02.fasta database with two different data processing pipelines and search engines: (1) the Mascot Distiller (version 2.5.1.0, Matrix Science) deconvoluted the MS/MS spectra and performed an MS/MS ion search, and (2) the PEAKS Studio (version 7) used a PTM algorism and performed a database search based on a de novo sequencing. These analyses were conducted until the beginning of 2018 and after excluding peptides derived from the keratin protein family, they resulted in the identification of 18,552 polypeptide sequences with a peptide identification false discovery rate (FDR) of 1%. All these acquisition data were already deposited in the ProteomeXchange Consortium via the PRIDE^23^ partner repository with the dataset identifier PXD003533. As described in the Methods section, we reanalysed these resources using the PEAKS Studio (version X), and a database search was performed against the SwissProt_2020_03.fasta with five variable PTMs in addition to the carbamidomethylation without using the PTM algorithm. This process resulted in the identification of 7,959 or 11,256 distinct native peptide sequences depending on FDRs of 0% or 1%, respectively^22^. We performed a bioinformatic analysis of this updated resource to select bioactive peptide candidates for chemical synthesis and functional validation using the criteria described in the Methods section. Synthesised peptides were tested for their high purity and liquid solubility using LC–MS/MS analysis and 135 peptides were confirmed to be appropriate for biological validation (Supplementary Table S1).

### Searching for novel polypeptides that elicit cellular responses

**Figure 1:**
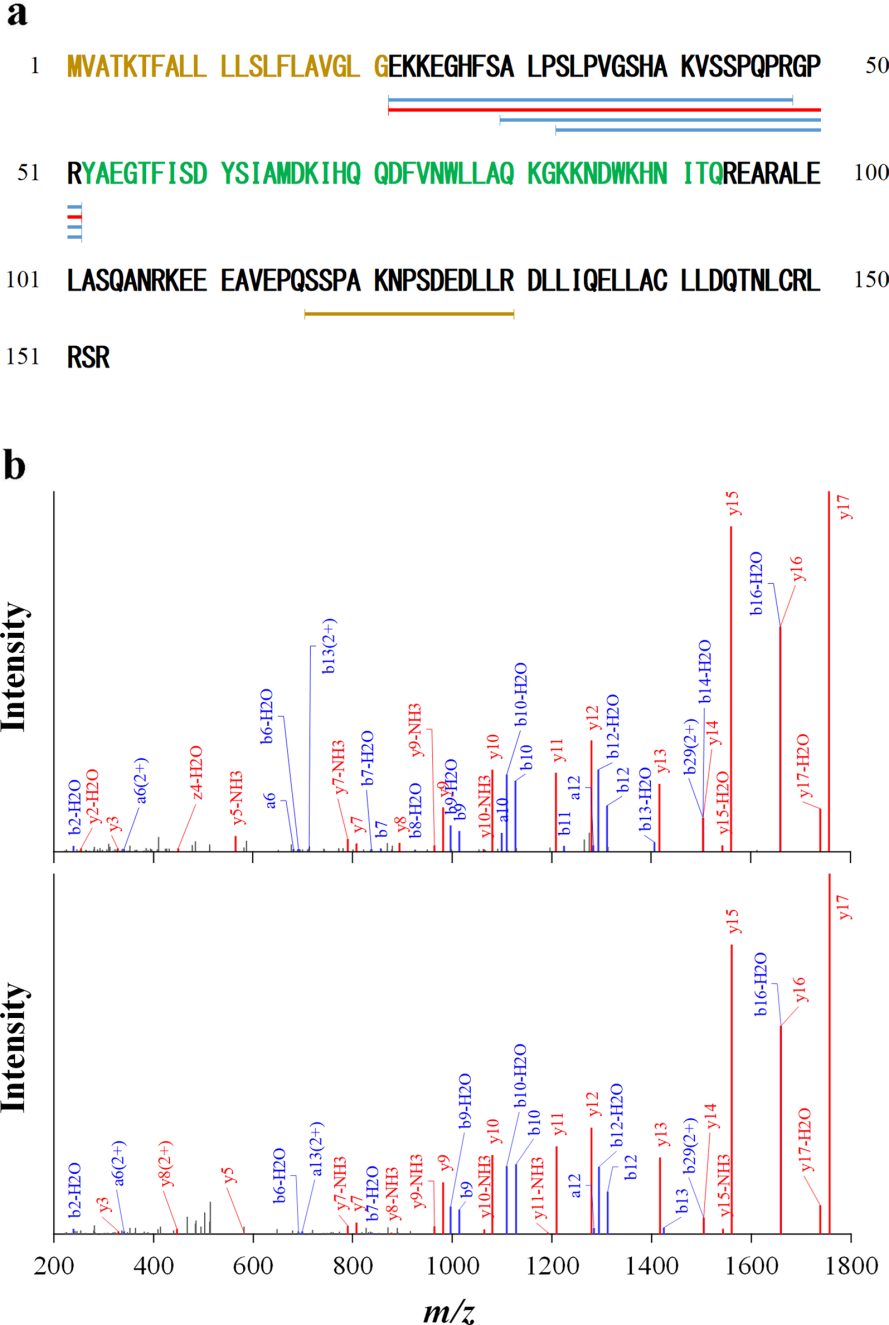
Peptidomic identification of proGIP-derived peptides from human plasma. (**a**) Sequence alignment of GIP_HUMAN[22–51] (red horizontal line) and other cleaved products of proGIP identified with an FDR of 0% (blue horizontal lines) or 1% (yellow horizontal lines) by our peptidomic analysis. The predicted signal peptide of the preproprotein is shown in yellow letters and authentic GIP peptide in green letters, respectively. (**b**) Annotated MS/MS fragmentation spectra for plasma GIP_HUMAN[22–51] filtered by peptidomic analysis and comparisons with those of the corresponding synthetic GIP_HUMAN[22–51] peptides. MS/MS spectra with sequence assignments of fragment ions corresponding to synthetic GIP_HUMAN[22–51] “EKKEGHFSALPSLPVGSHAKVSSPQPRGPR” with a *m/z* 530.9565 (*z* = 6; upper panel) are compared with those of endogenous peptides (lower panel) to confirm the putative identification. The lower panel shows the MS/MS spectrum obtained by LC–MS/MS analysis in Fig. 5b with a retention time of 10.82 min. MS/MS spectra were deconvoluted into singly charged ions from the observed spectra and peaks were assigned theoretical *m/z* values for fragment ions. The annotations of the identified matched N-terminal-containing ions are shown in blue and the C-terminal-containing ions in red. The *m/z* differences between theoretical and observed values for most assigned peaks were less than 0.01 Da.

**Figure 2:**
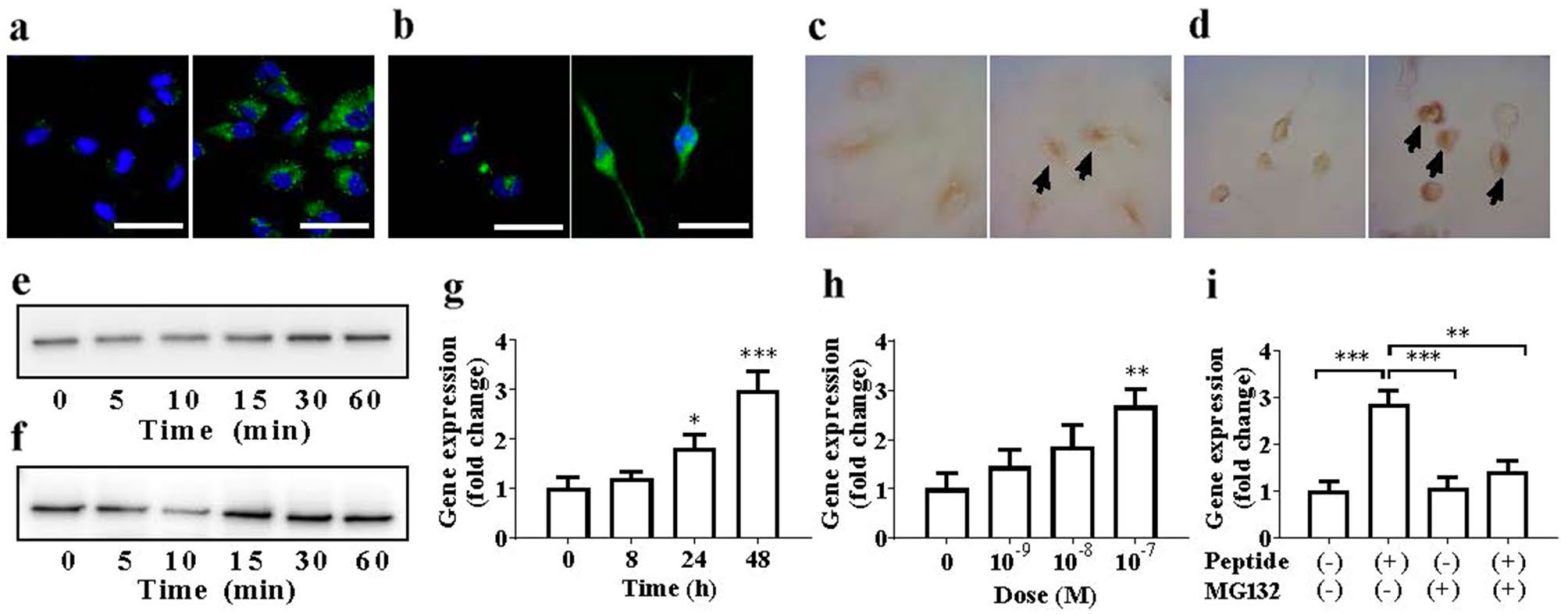
Cellular responses to GIP_HUMAN[22–51]. (**a, b**) Confocal laser-scanning microscopy images of the fluorescent GIP_HUMAN[22–51] peptides bound to cultured cells. Growing HAoECs (**a**) or THP1-derived macrophages deprived of serum for 16 h (**b**) were overlaid without (left panels) or with 10^–6^ M FAM-GIP_HUMAN[22–51] (right panels) for 30 or 5 min, respectively. Cells were washed, fixed, nuclei counterstained with DAPI (blue) and the cell surface-bound green fluorescence visualised. Scale bar represents 50 μm. (**c, d**) Nuclear translocation of NF-κB. HAoECs (**c**) or THP1-derived macrophages (**d**) were incubated without (left panel) or with 10^–6^ M GIP_HUMAN[22–51] for 60 min, and immunocytochemical staining was performed using an NF-κB p65 subunit antibody to detect its nuclear translocation. (**e, f**) Degradation of IκB-α. HAoECs (**e**) or THP1-derived macrophages (**f**) were stimulated with 10^–7^ M GIP_HUMAN[22–51] for the indicated times and subjected to western blot analysis using the anti-IκB-α antibody to assess the time-course of IκB-α degradation. The panels show the cropped blots and the full-length blots are presented in the Supplementary Information. (**g–i**) Upregulation of MMP8 gene expression by GIP_HUMAN[22–51]. HAoECs were incubated with 10^–7^ M GIP_HUMAN[22–51] for the indicated times (**g**) or with indicated doses for 48 h (**h**), and MMP8 mRNA and β-actin levels were quantified. Data represent the fold changes (mean ± S.E.M) of MMP8 mRNA copies relative to β-actin mRNA (*n* = 5). *p < 0.05, **p < 0.01, ***p < 0.001 compared with vehicle. (**i**) HAoECs pre-treated with or without MG132 (500 nM) for 30 min were incubated with 10^–7^ M GIP_HUMAN[22–51] and MG132 (100 nM) for 24 h and MMP8 and β-actin mRNA levels were quantified. **p < 0.01, ***p < 0.001, compared with vehicle. The relative mRNA levels are shown as fold changes (mean ± S.E.M; *n* = 5).

Of the 135 synthetic peptides, we completed functional screening of 129 peptides in three types of cultured human cells, including the human monocytic leukaemia cells (THP1) induced to differentiate into macrophages, aortic endothelial cells (HAoECs), and aortic smooth muscle cells (HAoSMCs). We found that a 30-amino-acid peptide derived from GIP (Fig. 1a,b), EKKEGHFSALPSLPVGSHAKVSSPQPRGPR (GIP_HUMAN[22–51], monoisotopic mass 3179.6951), caused an increase in intracellular free Ca^2+^ levels ([Ca^2+^]_i_) in the THP-1-induced macrophages. The ability of GIP_HUMAN[22–51] to bind and elicit intracellular responses was examined, using the three types of cultured human cells. These cells were incubated with the peptide labelled with 5-carboxyfluorescein at the N-terminus (FAM-GIP_HUMAN[22–51]) to determine whether GIP_HUMAN[22–51] was bound to the intact cell surface. Confocal immunofluorescence microscopy revealed the presence of FAM-GIP_HUMAN[22–51] on the surface of the HAoECs (Fig. 2a) and THP1-derived macrophages (Fig. 2b), but not in the HAoSMCs. Of the remaining 128 peptides, two suprabasin-derived peptides elicited significant cellular responses in the HAoSMCs^22^. Three other peptides induced marginal increases in [Ca^2+^]_i_ in the HAoECs; however, their detailed biological activity has not subsequently been investigated.

### Activation of NF-κB by GIP_HUMAN[22–51]

The HAoECs and macrophage-differentiated THP1 cells were treated with GIP_HUMAN[22–51] for different periods and immunostained with an antibody against the p65 subunit of the NF-κB. GIP_HUMAN[22–51] increased the NF-κB immunoreactivity in the nuclei of both the HAoECs and THP1-derived macrophages within 60 min, indicating a nuclear translocation of the NF-κB (Fig. 2c,d). GIP_HUMAN[22–51] caused a rapid (within 5–15 min) and transient degradation of the IκB-α in the HAoECs and THP1-derived macrophages, which returned to baseline levels within 1 h (Fig. 2e,f). Next, we searched for genes upregulated by GIP_HUMAN[22–51] via NF-κB signalling in the HAoECs. GIP_HUMAN[22–51] induced the expression of the matrix metalloproteinase-8 (MMP-8) gene in a time- and dose-dependent manner (Fig. 2g,h). To determine the involvement of NF-κB signalling in the GIP_HUMAN[22–51]-mediated MMP-8 gene expression, we pretreated the HAoECs with a proteasome inhibitor, MG132, and stimulated them over a time course with GIP_HUMAN[22–51]. The inhibition of NF-κB by MG132 prevented the MMP-8 expression induced by GIP_HUMAN[22–51] (Fig. 2i). These results indicated the role of GIP_HUMAN[22–51] in NF-κB activation in vascular endothelial cells and macrophages.

Our human plasma peptidomic library contained four additional proGIP-derived peptides; two of these (GIP_HUMAN [30–-51] and GIP_HUMAN [33–51]) had FDRs of 0% and were fragments of GIP_HUMAN[22–51] (Fig. 1a, Supplementary Table S1). Neither peptide elicited cellular responses similar to that of GIP_HUMAN[22–51]. Thus, the N-terminal sequence of GIP_HUMAN[22–51] was considered to be responsible for the biological activity of GIP_HUMAN[22–51].

### Effect of GIP_HUMAN[22–51] infusions on atherosclerotic lesion progression

**Figure 3:**
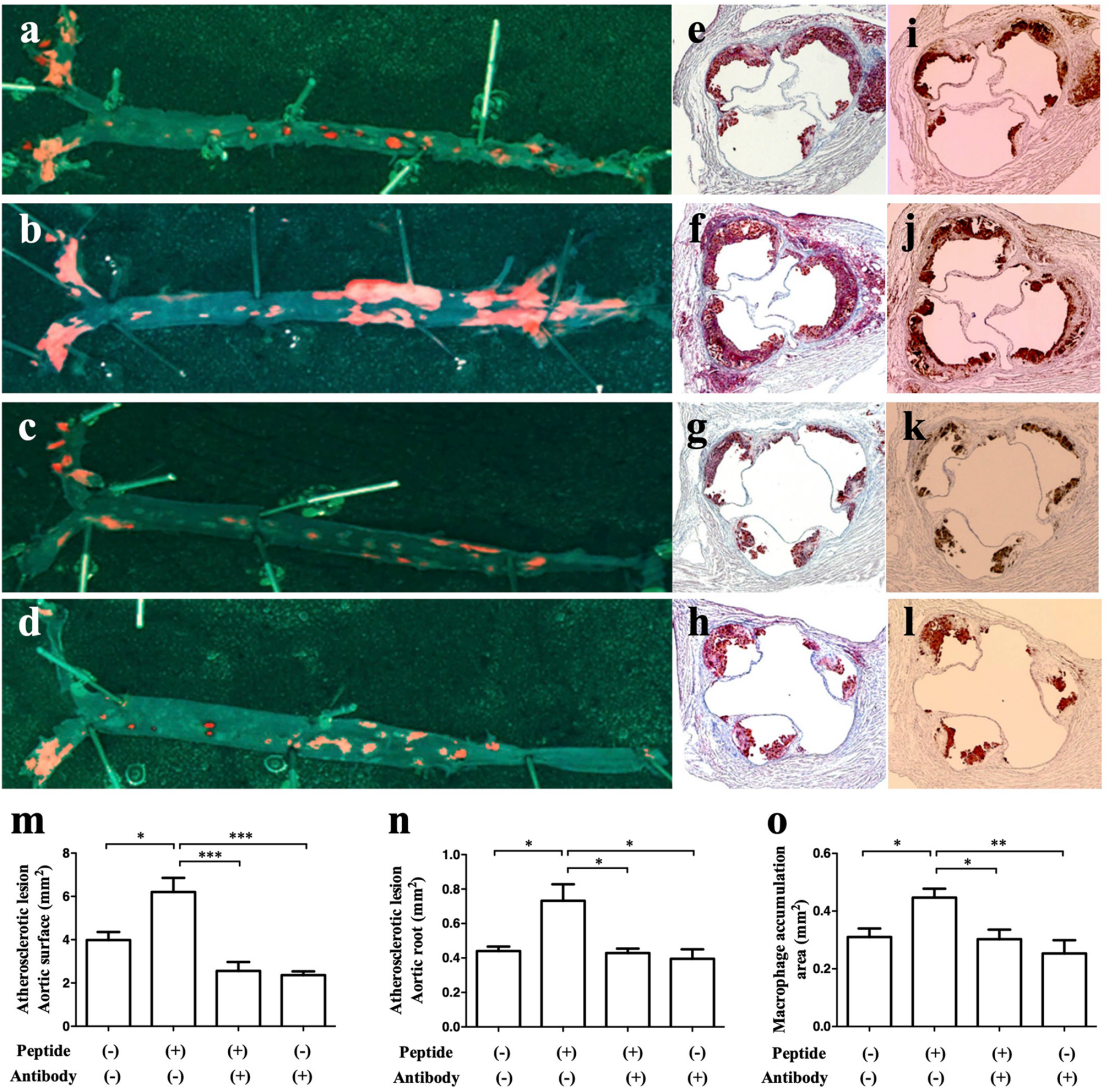
Proatherosclerotic effects of GIP_HUMAN[22–51] in *ApoE*^*−/−*^ mice. The 17-week-old *ApoE*^*−/−*^ mice were infused with saline alone (**a**, **e,** and **i**, vehicle), GIP_HUMAN[22–51] (**b**, **c**, **f**, **g**, **j,** and **k**, 0.6 nM/kg/h), and/or anti-GIP_HUMAN[22–51] IgG (**c**, **d**, **g**, **h**, **k,** and **l**, 1.4 μg/kg/h) by osmotic mini-pumps for 4 weeks. The aortic surface was stained with oil red O (**a**–**d**). Cross sections of the aortic root were stained with oil red O (**e**–**h**) or anti-MOMA-2 antibody (**i**–**l**) and counterstained with hematoxylin. Surface area of the atherosclerotic lesions (**m**), cross sectional area of the atherosclerotic lesion (**n**), and macrophage accumulation (**o**) are expressed as means ± SEM (*n* = 5–7). *p < 0.05, **p < 0.01, ***p < 0.001 compared with vehicle.

A sequence comparison of mouse and human proGIP proteins revealed that the N-terminal sequence of GIP_HUMAN[22–51] is highly homologous to the corresponding mouse GIP N-terminally extended putative peptide that was located immediately downstream of the signal peptide. Therefore, the in vivo effect of a continuous infusion of GIP_HUMAN[22–51] on atherosclerotic plaque formation in apolipoprotein E-deficient (*ApoE*^*−/−*^) mice was investigated. Two osmotic mini pumps were implanted subcutaneously and loaded with saline alone, saline-dissolved GIP_HUMAN[22–51], or anti-GIP_HUMAN[22–51] IgG. The continuous infusion commenced at 17 weeks of age and lasted until 21 weeks of age in all four groups of mice that were assigned to be infused with (1) saline and saline, (2) GIP_HUMAN[22–51] and saline, (3) GIP_HUMAN[22–51] and anti-GIP_HUMAN[22–51] IgG or (4) saline and anti-GIP_HUMAN[22–51] IgG. The daily food intake during the 4-week infusion period did not differ significantly between any two groups of mice (vehicle group: 3.57 ± 0.19 g/day; GIP_HUMAN[22–51] group: 3.51 ± 0.16 g/day; GIP_HUMAN[22–51] plus antibody group: 3.48 ± 0.19 g/day; antibody only group: 3.48 ± 0.22 g/day, n.s.). There were no significant differences in body weight among the four groups at any time points (Supplementary Fig. S1), nor were there any differences in serum chemistry measurements at the end of the 4-week infusion, except for an elevation of serum GIP levels in the GIP_HUMAN[22–51] plus anti-GIP_HUMAN[22–51] IgG group (Supplementary Table S2). After 4 weeks of infusion, the atherosclerotic lesions were evaluated according to the oil red O-stained aortic surface area (Fig. 3a–d), the aortic root area (Fig. 3e–h), and the anti-MOMA-2 antibody-stained aortic root area (Fig. 3i–l). GIP_HUMAN[22–51] increased the atherosclerotic lesion areas and atheromatous plaque formation significantly when compared with that of the vehicle infusions (Fig. 3m–o), whereas the combined infusions with the neutralising antibody inhibited the proatherosclerotic effects caused by GIP_HUMAN[22–51] (Fig. 3c,g,k).

**Figure 4:**
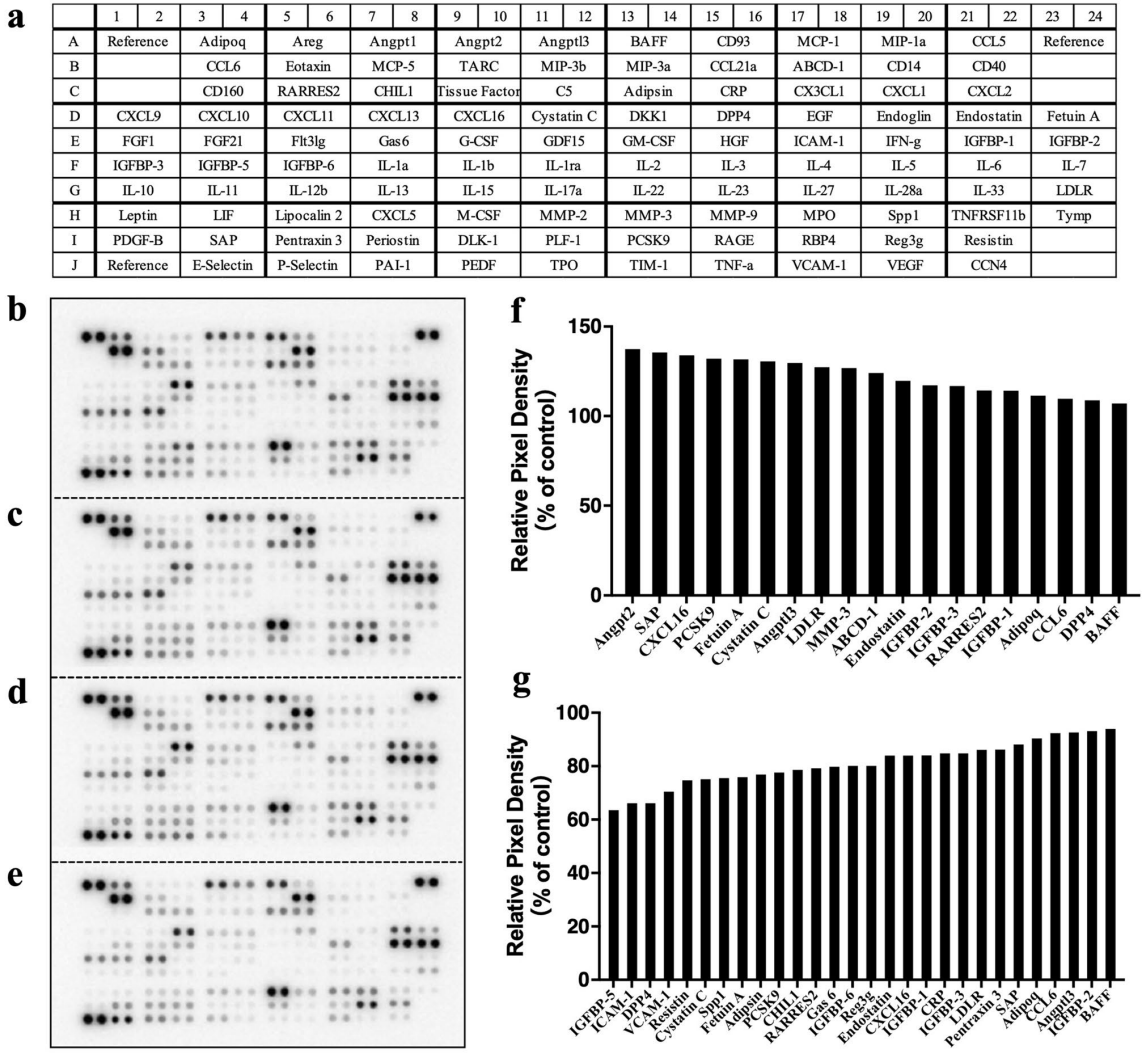
Cytokine array screening of mice serum infused with GIP_HUMAN[22–51] for 4 weeks. (**a**) Layout of antibody arrays and abbreviated names of the 111 cytokine/chemokine probes, adapted from the manufacturer’s information. (**b**–**e**) Complete hybridisation signals after probing with serum samples obtained from ApoE−/− mice infused for 4 weeks with saline alone (**b**), GIP_HUMAN[22–51] (**c**), combined infusion with GIP_HUMAN[22–51] and anti-GIP_HUMAN[22–51] IgG (**d**), or anti-GIP_HUMAN[22–51] IgG (**e**). (**f**, **g**) Quantified signal intensities of the respective serum proteins in mice infused with GIP_HUMAN[22–51] (**f**) or anti-GIP_HUMAN[22–51] IgG (**g**) in terms of 2-spot mean values relative to untreated experiments are shown.

To investigate whether chronic infusions of GIP_HUMAN[22–51] stimulated the production and/or release of bioactive substances, serum samples withdrawn from the *ApoE*^*−/−*^ mice after 4 weeks of infusions with or without GIP_HUMAN[22–51] or anti-GIP_HUMAN[22–51] IgG, were applied to protein antibody arrays that detected 111 mouse cytokines and chemokines (Fig. 4a). The representative blots generated by these analyses showed appreciable regulation by GIP_HUMAN[22–51] and anti-GIP_HUMAN[22–51] IgG (Fig. 4b–e). The relative signal intensities of a variety of protein levels induced by GIP_HUMAN[22–51] or anti-GIP_HUMAN[22–51] IgG versus the vehicle-treated control experiments were quantified (Fig. 4f,g). The infusions of GIP_HUMAN[22–51] increased the secretions of many proinflammatory and proatherosclerotic proteins, including angiopoietin-2, serum amyloid P (SAP), CXC chemokine ligand 16 (CXCL16), proprotein convertase subtilisin kexin type 9 (PCSK9), fetuin A and MMP-3, whereas the anti-GIP_HUMAN[22–51] IgG reduced the serum levels of the intercellular adhesion molecule-1 (ICAM-1), vascular cell adhesion molecule-1 (VCAM-1), fetuin A, PCSK9, CXCL16, C-reactive protein and SAP. These results indicated that GIP_HUMAN[22–51] was an endogenous inducer of various proinflammatory and proatherosclerotic proteins.

### Presence of GIP_HUMAN[22–51] in human plasma and organs

**Figure 5:**
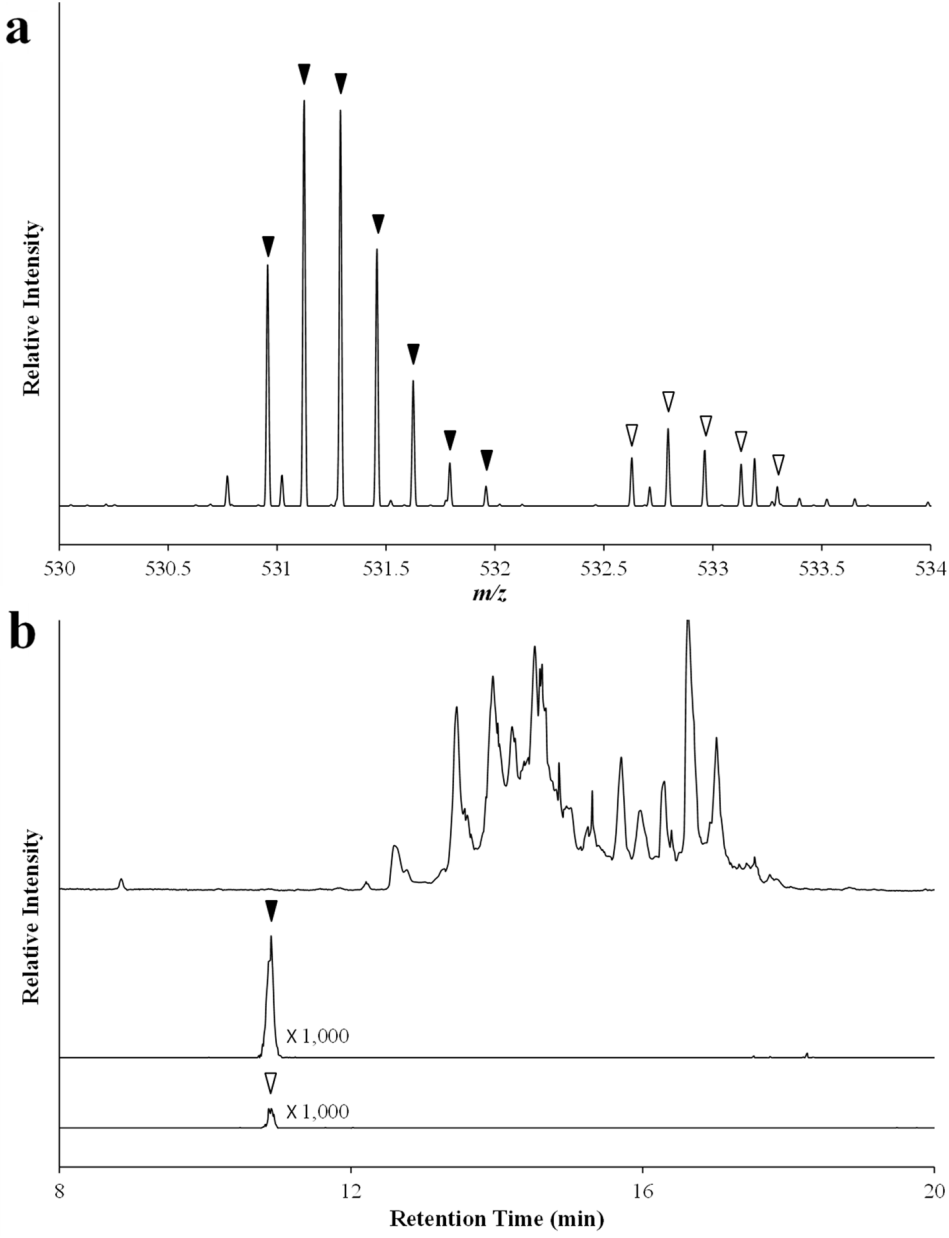
Quantitative analysis of the abundance of GIP_HUMAN[22–51] using LC–MS/MS. The stable isotope-labelled peptide was spiked into human plasma at the final concentration of 100 pM, enriched using the modified differential solubilisation method as described in the Methods and after desalting, the extracts derived from 10 μL plasma was analysed by LC–MS/MS. (**a**) MS spectrum of hexavalent ions observed at retention time 10.78–11.00 min (indicated by triangles in **b**). Closed triangles represent those derived from the endogenous GIP_HUMAN[22–51] (*m/z* = 530.9564, 531.1236, 531.2905, 531.4578, 531.6245, 531.7927, 531.9588) and open triangles from the synthetic stable isotope-labelled peptide (*m/z* = 532.6279*,* 532.7944*,* 532.9622*,* 533.1296*,* 533.2957). (**b**) Comparison of the total ion chromatogram (upper panel) and hexavalent ion XICs with ± 3 ppm mass window of the endogenous (middle panel) and stable isotope-labelled peptide (lower panel). Total XIC area, defined as the sum of three ion precursors; monoisotopic mass (M), XICs of the first isotope peak (M + 1) and the second isotope peak (M + 2), was used to extrapolate plasma GIP_HUMAN[22–51] levels.

To verify that GIP_HUMAN[22–51] was reproducibly detected according to the identified amino acid sequence in human plasma and to determine its accurate plasma levels, we synthesised a stable isotope-labelled GIP_HUMAN[22–51] and spiked the human plasma samples with a dilution series of this peptide prior to extraction to generate and extrapolate the extracted ion chromatogram (XIC) intensities using an ultra-high resolution mass liquid chromatography mass spectrometry method. The plasma concentration of GIP_HUMAN[22–51] extrapolated from the XICs generated by the respective endogenous peptides and the reference peptides was ~ 0.6 nM (Fig. 5). A raw data file of LC–MS/MS including MS spectrum and XIC (Fig. 5) and MS/MS spectrum (Fig. 1b, lower panel) has been deposited in the ProteomeXchange Consortium (http://proteomecentral.proteomexchange.org) via the jPOST partner repository (http://jpostdb.org)^24^ with the dataset identifier PXD026885 for ProteomeXchange and JPST001228 for jPOST.

**Figure 6:**
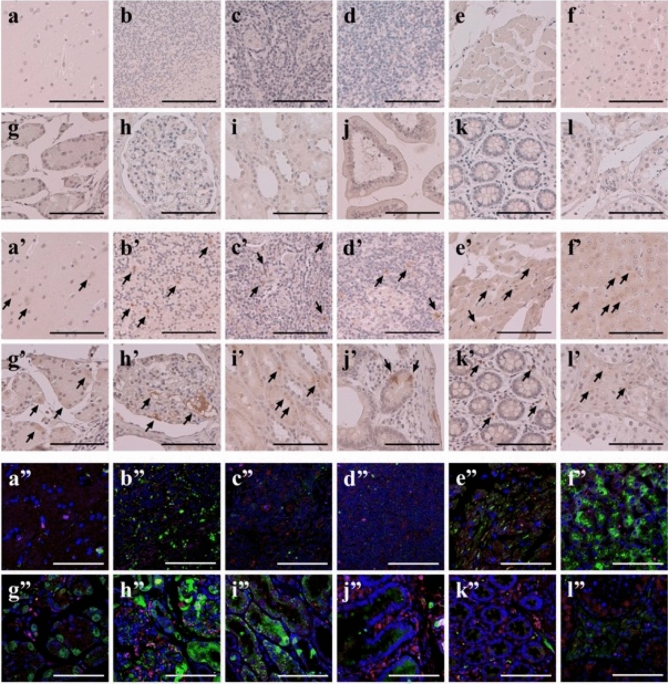
Systemic expression of GIP_HUMAN[22–51] and its negative colocalisation with GIP in major human organs. (**a–l**, **a**’–**l**’) Human tissue array sections were immunohistochemically stained with either IgG purified from preimmune rabbit serum (**a–l**) or anti-GIP_HUMAN[22–51] IgG at 1:500 dilution (**a**’–**l**’). Arrows indicate the positions of positive immunoreactivity. (**a”**–**l”**) Confocal microscopic images of immunofluorescent dual staining of GIP_HUMAN[22–51] and GIP. Human tissue array sections were double-stained with either the rabbit polyclonal anti-GIP_HUMAN[22–51] antibody (× 1000) or the mouse monoclonal anti-human GIP antibody (× 500). The red signals corresponding to the localisation of GIP were obtained with the Alexa Fluor 594 secondary antibody (× 1000) and the green signals representing GIP_HUMAN[22–51] were obtained with the Alexa Fluor 488 secondary antibody (× 500). The nuclei were counterstained with DAPI (blue). Overlay resulted in yellow signals indicative of colocalisation. (**a–a”**) cerebral cortex, (**b**–**b**”) cerebellum, (**c**–**c**”) tonsil, (**d**–**d**”) lymph node, (**e**–**e**”) heart, (**f**–**f**”) liver, (**g**–**g**”) stomach, (**h**–**h**”) kidney (glomerulus), (**i**–**i**”) kidney (tubule), (**j**– **j**”) small intestine, (**k**–**k**”) colon and (**l**–**l**”) testis (scale bar: 100 μm).

To investigate the systemic expression of GIP_HUMAN[22–51], a healthy human tissue microarray was stained using a specific polyclonal anti-GIP_HUMAN[22–51] IgG. Immunohistochemistry with IgG purified from preimmune rabbit serum did not detect any signal (Fig. 6a–l). Conversely, immunoreactive GIP_HUMAN[22–51] was abundant in the cerebellum, liver, stomach, kidney, heart, small intestine, and colon, and less in the cerebral cortex, tonsils, lymph nodes, and testes (Fig. 6a’–l’), and undetected in lung, pituitary, spleen, pancreas, adrenal gland, spine, thyroid, bone marrow, fallopian tube, skin, thymus, uterus, bladder, ovary and prostate. GIP_HUMAN[22] is biosynthesised theoretically, concomitantly with GIP from their common precursor protein, and GIP is expressed abundantly in a limited number of organs, such as the small intestine and duodenum. Therefore, we investigated whether GIP and GIP_HUMAN[22–51] peptides were co-localised within cells by co-staining the two peptides. Confocal immunofluorescence microscopy revealed a distinct GIP and GIP_HUMAN[22–51] expression within each type of tissue (Fig. 6a”–l”). These results indicate differential intracellular production and/or localisation of the GIP_HUMAN[22–51] and the GIP peptides.

## Discussion

We described the identification of a novel peptide hormone, GIP_HUMAN[22–51], using native peptide resources generated by plasma peptidomics. We completed functional screening of 129 synthetic peptides selected in silico using cultured human macrophages and vascular cells. Since GIP_HUMAN[22–51] activated NF-κB signalling in the HAoECs and the macrophages, its proatherogenic activity was investigated using *ApoE*^*−/−*^animal models. GIP_HUMAN[22–51] accelerated the development of atherosclerotic lesions without affecting body weight and food intake, whereas combined infusions of the anti-GIP_HUMAN[22–51] antibody inhibited the atheromatous plaque formation and macrophage accumulation induced by GIP_HUMAN[22–51]. GIP_HUMAN[22–51] upregulated the serum levels of many important proatherosclerotic and proinflammatory proteins that are transcriptionally upregulated by NF-κB, whereas the neutralising endogenous GIP_HUMAN[22–51] with the anti-GIP_HUMAN[22–51] antibody suppressed these levels. Thus, GIP_HUMAN[22–51] revealed a novel endogenous proatherogenic peptide that activates NF-κB signalling.

GIP_HUMAN[22–51] is located immediately downstream of the signal peptide of the preproGIP and is theoretically biosynthesised simultaneously upon generation of the mature GIP from its common precursor, proGIP. Signal peptides of preproproteins are cleaved cotranslationally^25,26^, whereas the prohormone convertase (PC) 1/3 flanks the C-terminus of GIP_HUMAN[22–51] to generate both GIP_HUMAN[22–51] and GIP from proGIP^5^. The endogenously processed GIP_HUMAN[22–51] has a C-terminal PC consensus motif that can be removed by carboxypeptidase E, which is a common feature of most neuropeptides and peptide hormones^27^. However, the LC–MS/MS analysis did not detect GIP_HUMAN[22-50], which does not contain a C-terminal arginine residue. The absence of GIP_HUMAN[22–50] in our repetitive LC–MS/MS analysis was not likely to be caused by a reduced ionisation efficiency due to the loss of the arginine because GIP_HUMAN[22–50] has four positively charged residues, that is, three lysines and one arginine. In addition to GIP_HUMAN[22–51], our current LC–MS/MS analysis identified GIP_HUMAN[30–51] and GIP_HUMAN[33–51] in human plasma with the highest confidence probability of an FDR of 0%. These two plasma peptides did not exert any appreciable biological activity when compared with that of GIP_HUMAN[22–51], suggesting that the effect of GIP_HUMAN[22–51] required its N-terminal sequences.

The potential implications of GIP_HUMAN[22–51] in the pathophysiology of human diseases is supported by its presence in the human peripheral circulation. One of the major obstacles in the study of plasma peptides is the difficulties associated with their precise quantification. Our LC–MS/MS measurements by high resolution Orbitrap MS used plasma pre-spiked with synthetic stable-isotope labelled peptides to exclude any interference associated with the plasma extraction^28^. Further, we used the sums of the XIC areas of the three ion precursors generated by the endogenous and synthetic peptides to ensure accuracy in the quantification^29,30^ (Fig. 5). This yielded a human plasma concentration of approximately 0.6 nM. The levels were higher than those of the major pituitary, pancreatic and vasoactive peptide hormones and comparable with other hormones, such as cortisol, and some growth factors.

Systemic immunohistochemical analysis using a normal human tissue microarray revealed an abundant expression of GIP_HUMAN[22–51] in several digestive organs and kidneys. Conversely, GIP is synthesised throughout the small intestine, mainly in the duodenum and proximal jejunum in the enteroendocrine K cells^31,32^, and is secreted in response to nutrient ingestion, especially glucose or fat^33,34^. Our confocal immunofluorescent microscopy results showed the concomitant expression of GIP and GIP_HUMAN[22–51] at tissue levels; however, these two peptides were not colocalised at the cellular level, despite both peptides being, in principle, biosynthesised simultaneously from the proGIP. GIP_HUMAN[22–51] is considered to act on its own unidentified receptor distinct form the GIP receptor, because GIP and GIP_HUMAN[22–51] exert totally distinct responses in both cultured cells and animal models. In a previous study using *ApoE*^*-/-*^ mice, GIP induced antiatherogenic effects by activating cAMP via the GIP receptor^35^, while in the current study, GIP_HUMAN[22–51] caused NF-κB activation and contrasting proatheroslcerotic effects. Several classical bioactive peptides are derived from the same precursor proteins as a result of tissue-specific posttranslational processing mechanisms and they show distinct biological activities and systemic expression profiles. For example, the prohormone convertase 2 expressed in the pancreatic α-cells converts proglucagon into glucagon, while prohormone convertase 1/3 in small intestinal enteroendocrine L-cells processes proglucagon into GLP-1^36^. Other examples include proopiomelanocortin-derived peptides in the anterior pituitary^37,38^ and salusin-α/salusin-β in systemic tissues^15^. GIP/GIP_HUMAN[22–51] is another pair that shows distinct biosynthetic mechanisms and biological activities.

Identification of plasma bioactive peptides and disease biomarkers is challenging because these peptides usually exist at trace levels, while plasma contains a considerable dynamic range of high-molecular-weight proteins. This study demonstrated that the use of our plasma native peptide library identified using our improved peptidomics technology, enabled an easier identification of a novel bioactive peptide than conventional methods. Synthetised peptides of the identified sequences can be tested for biological responses using experiments that detect the desired activity. These peptides can also be tested to identify ligands of orphan GPCRs. The non-biased peptidomic information generates a human “orphan ligand” library available for the discovery of novel bioactive peptides and disease biomarkers applicable to various scientific fields. Our results will facilitate the ultimate application of peptidomics techniques in biomedical fields and further promote innovations in the health science industry.

Our annotation of the identified peptide, GIP_HUMAN[22–51], may lead to a misunderstanding of the amino acid position as the authentic active GIP peptide is often called GIP(1–42) to distinguish it from its inactive form GIP(3–42), indicating that this peptide consists of 42 amino acids. However, we have proposed the use of the UniProt entry names with their amino acid positions, such as GIP_HUMAN[22–51], rather than assigning new names to each new bioactive peptide discovered, because the current peptidome has already revealed the presence of a large number of fragmented peptides of many classical proteins/peptides, which may facilitate the discovery of multiple new bioactive peptides^22^ and biomarkers derived from a single proprotein. Classical bioactive peptides and biomarkers can also use such aliases in the event that the discovery of their brotherhood peptides can cause confusion. Authentic active and inactive incretin hormones, GIP(1–42) and GIP(3–42), can be called GIP_HUMAN[52–93] and GIP_HUMAN[54–93], respectively, to compare their amino acid positions in the entire preproGIP protein.

In conclusion, this was the first study to discover a novel proatherosclerotic peptide by using the native peptide resources identified by the human plasma peptidome. This outcome was made possible by our improved technology that efficiently enriched plasma low-molecular-weight, low-abundance native peptides, enabling their identification by LC–MS/MS analysis. Human plasma peptidomics holds significant promise as a powerful revolutionary tool for identifying endogenous bioactive peptides and peptide biomarkers that may detect, diagnose, and treat diseases.

## Methods

### Database searching of LC–MS/MS acquired peptidomic resource

LC–MS/MS acquisition data obtained up until the beginning of 2018 were initially searched against the SwissProt_2015_02.fasta database (selected for *Homo sapiens*; 20,199 entries) using two different data processing pipelines and search engines, Mascot (Matrix Science, London, UK) and PEAKS Studio (Bioinformatics Solutions, Waterloo, Canada)^22^. In the Mascot workflow, raw MS and MS/MS data files were processed using Mascot Distiller (version 2.5.1.0, Matrix Science). Data processing included peak picking, de-isotoping and charge deconvolution of fragment ions. The resulting peak list files were searched using the Mascot search engine (version 2.4.1). In the PEAKS Studio workflow, the PEAKS Studio (version 7.0) was used to perform peak picking, de-isotoping, charge deconvolution of fragment ions and a de novo peptide sequencing-based database search from the MS and MS/MS spectra of peptides. The PTM algorithm of the PEAKS Studio was applied to identify variable modifications and substitutions^39–41^. The FDR was set as 1%. All the peptidomic sequencing data were deposited into the ProteomeXchange Consortium via the PRIDE^42^ partner repository with the dataset identifier PXD003533.

In our initial analyses of the peptides deposited in the peptidomic database, we used 485 built-in modifications in the PTM search of the PEAKS, which hampered the PTM accuracy. Furthermore, a native peptide database search was performed using the Mascot MS/MS ion search, in which peptides longer than 30 amino acids may show high scores despite having poor MS/MS spectra. Therefore, we reanalysed the entire peptide database using the SwissProt_2020_03.fasta database (selected for *Homo sapiens*; 20,365 entries) with the PEAKS database search algorithm (Bioinformatics Solutions, Waterloo, Canada). The PEAKS Studio (version X) performed peak picking, de-isotoping, charge deconvolution of fragment ions and a de novo peptide sequencing-based database search from the MS and MS/MS spectra of peptides. The search parameters were: enzyme, no enzyme; fixed modification, carbamidomethyl (C, only for samples with reductive alkylation); variable modifications, acetyl (N-term, K), amidated (C-term), pyro-glu from Q (Q), oxidation (M), carbamidomethyl (N-terminal, only for samples with reductive alkylation); peptide ion mass tolerance, 6 ppm; fragment ion mass tolerance, 0.02 Da. The FDR was set at 1%. The results of the analysis using this updated database and the PEAKS search algorithm were used for the subsequent exploration of the bioactive peptides.

### Selection of peptides for chemical synthesis and dissolution of synthetic peptides

The peptides for synthesis and the biological function analysis were selected in silico based on: (1) being uniquely assigned to a precursor protein family; (2) being assigned a gene name or a gene name of a protein family; (3) being encoded by secretory proteins as defined by the SwissProt keywords; (4) having potential extracellular release predicted by PSORT, a bioinformatics tool used for the prediction of protein localisation sites in cells^43^; (5) being identified with an FDR of 1%; (6) having amino acid lengths of 5–38 residues; (7) having no substitutions or modifications except for C-terminal amidation; and (8) having no cysteine residues. For the initial round of chemical synthesis, 146 sequences were selected and synthesised (Scrum Inc., Tokyo, Japan), reconstituted to 2–10 × 10^−6^ M with 10% acetonitrile/0.1% trifluoroacetic acid and then subjected to an LC–MS/MS analysis to confirm the purity and solubility.

### Production and purification of peptides antibodies

The specific polyclonal antibody against GIP_HUMAN[22–51] was raised and purified as described previously^20,44^, with the following modifications: two micrograms of [Cys^0^]-PEG-EKKEGHFSALPSLPVGSHAKVSSPQPRGPR were chemically synthesised and approximately half was pre-treated with a protein crosslinking and fixation reagent, mixed with the remaining respective 1 μg of untreated peptide, coupled to a maleimide-activated mariculture keyhole limpet hemocyanin (Pierce) and immunised on days 1, 15, 29, 43 and 57 into Japanese white rabbits. Blood was collected prior to the first injection and on days 36, 50 and 64 post-injection. The antibody titer was determined using ELISA. The polyclonal antisera were purified using a Melon Gel IgG Spin Purification Kit (Thermo Fisher Scientific, Waltham, MA, USA), which removed the non-relevant proteins often present in high abundance^20^.

### Cell culturing

The human aortic endothelial cells (HAoECs) and human aortic smooth muscle cells (HAoSMCs) were purchased from Promocell (Heidelberg, Germany) and cultured using the appropriate medium and supplements recommended by the supplier. Successive experiments were performed with passage of four to six cultures. The THP1 was obtained from the Riken Cell Bank (Ibaraki, Japan). The THP1 cells were grown in a RPMI1640 medium containing 10% foetal bovine serum for 2–3 days and then incubated with 500 ng/mL phorbol-12-myristate-13-acetate for 24 h to facilitate the differentiation into macrophages.

### Determination of the intracellular free Ca^2+^ concentration [Ca^2+^]_i_

The HAoECs were placed in non-coated 96-well black plates with clear bottoms, deprived of serum for 16 h, and incubated with a fluo-4-acetoxymethyl ester (Fluo 4-AM; Dojindo Molecular Technologies, Kumamoto, Japan) at 37 °C for 30 min in Hank’s balanced salt solution (HBSS). Fluo 4-AM-loaded cells were washed three times with HBSS, applied with dissolved synthetic peptides and read at an excitation wavelength of 485 nm and emission wavelength of 535 nm using a Powerscan HT (BioTek Instruments Inc., Winooski, VT, USA) at the indicated times^15,45^.

### Cell surface binding of peptides

The THP1-derived macrophages deprived of serum for 16 h and the growing HAoECs were incubated for 5–60 min after the addition of 10^–6^ M FAM-labelled GIP_HUMAN[22–51]. The cells were washed three times with phosphate-buffered saline (PBS) and fixed with 4% paraformaldehyde for 15 min at room temperature. The nuclei were counterstained using DAPI Fluoromount-G (SouthernBiotech, Birmingham, AL, USA) and the fluorescence signals were photographed using an LSM710 confocal microscope (Carl Zeiss, Jena, Germany), as described previously^46,47^.

### Immunocytochemistry staining

To assess the NF-κB nuclear translocation, the THP1-derived macrophages and HAoECs grown in a 4-well Slide&Chamber (Watson Bio Lab, Tokyo, Japan) for 2–3 days were washed twice with PBS and incubated with GIP_HUMAN[22–51] (10^–7^ M) for 60 min. The cells were fixed with 4% paraformaldehyde for 15 min, blocked with Blocking One (Nacalai Tesque Inc., Kyoto, Japan), incubated for 60 min with an antibody against p65 (1:500, Santa Cruz Biotechnology, Inc., CA, USA) at room temperature and incubated for 30 min with the biotinylated goat anti-rabbit antibody (1:3000, Vector Laboratories Inc., Burlingame, CA, USA). The avidin–biotin-peroxidase complex was formed by using the Vectastain ABC Kit (Vector Laboratories Inc.) and visualized by using the DAB peroxidase substrate kit (Vector Laboratories Inc.).

### Immunoblotting

Western blotting was performed as described previously^46,48^. The subconfluent HAoECs and THP1-derived macrophages grown in 6-well plates were incubated with GIP_HUMAN[22–51] for the indicated times, washed twice with ice-cold PBS, solubilised in RIPA Lysis and Extraction Buffer (Thermo Fisher Scientific) containing a protease inhibitor cocktail (1:100, Thermo Fisher Scientific), sonicated, and centrifuged at 10,000 × *g* for 10 min at 4 °C. The supernatant was collected and the protein concentrations were measured. The extracted protein samples were loaded onto 4%–20% gradient polyacrylamide gels (Mini-PROTEAN TGX Gels, Bio-Rad Laboratories, Irvine, CA, USA) and transferred to PVDF membranes (Trans-Blot Turbo Mini PVDF Transfer Packs, Bio-Rad Laboratories). After blocking with Blocking One (Nacalai Tesque Inc.), the membranes were incubated with the anti-IκB-α antibody at a 1:3000 dilution (ab32518, Abcam, Cambridge, MA, USA) overnight at 4 °C, washed extensively, and then incubated with the peroxidase-conjugated goat anti-rabbit secondary antibody at 1:5000 dilution (Bio-Rad Laboratories) for 1 h at room temperature. The protein bands were detected using ECL Prime (GE Healthcare, Tokyo, Japan). The signals of each blot were visualised and analysed quantitatively using ImageQuant LAS 4000 (GE Healthcare)^47^.

### Real-time RT-PCR

Total RNA was harvested using the TRIzol reagent (Invitrogen, Carlsbad, CA, USA) from the HAoECs grown in 6-well plates treated with or without GIP_HUMAN[22–51], reverse transcribed with the PrimeScript RT Master Mix (Takara Bio Inc., Shiga, Japan) and quantified using a C1000 Touch Thermal Cycler and CFX Real-Time PCR Detection Systems (Bio-Rad Laboratories) and the Kapa Syber Fast qPCR Master Mix (Kapa Bio Systems Inc., Cape Town, South Africa) as described previously^47^, but with the following modifications: after the reverse transcription, the reaction mixtures were denatured at 94 °C for 3 min followed by 40 cycles of PCR at 94 °C for 10 s and 60 °C for 30 s. The data were expressed as *C*t values and used to determine Δ*C*t values. The human MMP8 mRNA was amplified using synthetic oligomers (MMP8: forward primer 5′-tctgcaaggttatcccaagg-3′, reverse primer 5′-gctccatgaattgtctttggt-3′; β-actin: forward primer 5′-attggcaatgagcggttc-3′, reverse primer 5′-ggatgccacaggactcca-3′) synthesised by Eurofins Genomics (Tokyo, Japan). The relative MMP8 gene expression level versus the reference β-actin expression level as the housekeeping gene was calculated using the 2^−ΔΔ*CT*^ method equipped in software packages for PCR Detection Systems^49^.

### In vivo analysis of atherosclerosis lesion development

Twenty-four *ApoE*^*−/−*^ 9-week-old mice purchased from Sankyo Labo Service (Tokyo, Japan) were maintained under controlled temperatures (22–25 °C) with free access to standard rodent chow (Labo MR Stock, NOSAN, Yokohama, Japan) for 4 weeks. At 13 weeks of age, the mice were fed the High-Fat-Diet (D12451, Oriental Yeast Co., Ltd., Tokyo, Japan) containing 45% fat. At 17 weeks of age, two micro-osmotic pumps (Alzet^Ⓡ^ Model 1002, Durect Corp., Cupertino, CA, USA) were implanted subcutaneously into the dorsum of mice that were anaesthetised with isoflurane in order to chronically infuse GIP_HUMAN[22–51] and/or its neutralising antibody. The pumps were replaced after 2 weeks. The mice were assigned into the following four groups with two pumps loaded with: 1) saline only (vehicle); 2) GIP_HUMAN[22–51] and saline; 3) saline and anti-GIP_HUMAN[22–51] IgG; and 4) GIP_HUMAN[22–51] and anti-GIP_HUMAN[22–51] IgG. The pumps containing GIP_HUMAN[22–51] or anti-GIP_HUMAN[22–51] IgG delivered 0.6 nmol/kg/h or 1.4 μg/kg/h of peptide or antibody dissolved in saline, respectively. After 4 weeks, mice were anaesthetised with isoflurane and, after blood collection from the descending vena cava, transcardially perfused with PBS and then with 4% paraformaldehyde^35^. The entire length of the aorta was carefully dissected from the root to the abdominal area, and cross sections of the aortic roots and the longitudinally exposed lumen surface were stained with oil red O for evaluation of the atherosclerotic plaques^35^. To visualise macrophage infiltration into the aortic wall, cross-sections of the aortic roots were stained with the anti-mouse MOMA-2 antibody (Chemicon International Inc., Temecula, CA, USA). An image analyser (NIH Scion Image, Frederick, MD, USA) was used to measure the areas of the aorta with atherosclerotic lesions.

Using samples obtained at the end of the infusion experiments, the serum levels of glucose (Glucose C-test Wako, Wako, Osaka, Japan), total cholesterol (Cholesterol E-test Wako, Wako), HDL-cholesterol (HDL Cholesterol E-test Wako, Wako), triacylglycerol (LabAssay Triglyceride, Wako), GIP (Mouse Gastric Inhibitory Polypeptide (GIP) (total) ELISA kit, Merck, Millipore, Billerica, MA, USA) and insulin (Ultra Sensitive PLUS Mouse Insulin ELISA Kit, Morinaga, Yokohama, Japan) were measured according to the manufacturers’ protocols.

### Antibody array analysis

Two-hundred-microliter serum samples obtained from mice infused with or without GIP_HUMAN[22–51] or anti-GIP_HUMAN[22–51] IgG for 4 weeks were applied to the Mouse XL Cytokine Kit (ARY028, R&D Systems, Minneapolis, MN, USA) and assayed according to the manufacturer’s instructions. Cytokine array signals were detected using the ImageQuant LAS 4000 (GE Healthcare)^47^ and quantified using ImageJ software (http://rsb.info.nih.gov/ij/^50^. Values from duplicate spots were averaged and the relative signals obtained from the serum of mice treated with GIP_HUMAN[22–51] or anti-GIP_HUMAN[22–51] IgG were compared with the control serum samples that were obtained from vehicle-treated mice.

### Peptide extraction

Blood samples were collected from healthy volunteers into vacutainers containing Na_2_-EDTA and immediately separated in a refrigerated centrifuge at 1,000 g for 20 min. Aliquots were immediately flash-frozen in liquid nitrogen and stored at − 80 °C until processing. The following stable isotope-labelled peptide was synthesised by Scrum Inc. using L-phenylalanine-N-9-fluorenylmethoxycarbonyl (^13^C_9_, 98%; ^15^ N, 98%): SI-GIP_HUMAN[22–51], EKKEGHFSALPSLPVGSHAKVSSPQPRGPR, with the underlined residue containing a stable isotope. The stable isotope-labelled peptide was spiked into human plasma at the final concentration of 100 pM, enriched using the modified differential solubilisation method, as described previously^28^, but with the following modifications. A 50-μL plasma sample was diluted 1:2 with 100 μL denaturing solution (7 M urea, 2 M thiourea and 20 mM dithiothreitol), slowly dropped into 2 mL ice-cold acetone, with stirring at 4 °C for 1 h and then centrifuged at 19,000 g for 15 min at 4 °C. The precipitate was resuspended in 1 mL 80% acetonitrile containing 12 mM HCl, mixed at 4 °C for 2 h and centrifuged again at 19,000 g for 15 min at 4 °C. The low molecular weight peptides fraction in the supernatant was lyophilised. The peptides dissolved in 80 uL Invitrosol LC/MS Protein Solubilizer (Thermo Fisher Scientific) were desalted using StageTips filled with Empore C18 sealant (3 M, MN, USA)^51^. The peptides were eluted with 40 μL 80% acetonitrile containing 0.1% trifluoroacetic acid. The sample solutions were then lyophilised and redissolved in 20 μL 3% acetonitrile (ACN) and 0.1% formic acid (FA).

### Measurements of plasma peptide levels using stable isotope-labelled peptides

Peptide extracts were analysed using a quadrupole Orbitrap benchtop mass spectrometer, Q-Exactive (Thermo Fisher Scientific), equipped with an EASY-nLC 1000 system (Thermo Fisher Scientific). The peptides were injected directly onto an analytical column (C18, particle diameter 3 µm, 75 μm i.d. × 125 mm, Nano HPLC Capillary Column, Nikkyo Technos, Tokyo, Japan) and then separated with a gradient composed of solvent A (0.1% FA) and solvent B (0.1% FA and 90% ACN) (0–20 min, 5–32% B; 20–26 min, 32–55% B; 26–27 min, 55–95% B; 27–30 min, 95% B) at a flow rate of 300 nL/min. MS spectra were collected over an m/z range of 350 to 900 at a resolution of 70,000 at 200 m/z to set an automatic gain control (AGC) target of 1 × 10^6^. The 12 most-intense ions with charge states of 2 + to 6 + that exceeded an intensity of 1.3 × 10^5^ were fragmented in data-dependent mode via collision-induced dissociation with a normalised collision energy of 27%. Tandem mass spectra were acquired on the Orbitrap mass analyser with a mass resolution of 35,000 at 200 m/z to set an AGC target of 2 × 10^5^. The dynamic exclusion time was set to 30 s. The plasma concentrations were extrapolated from the XICs generated using the respective endogenous peptides and the corresponding spiked stable isotope-labelled peptides^30,52^.

### Immunohistochemistry

A human healthy tissue microarray, MNO341 (US Biomax Inc., Rockville, MD, USA), which containsed biospecimens of 33 tissue types mostly from surgical resectiosn, was used to examine the systemic distribution of each peptide expression. The tissue preparations were deparaffinised, rehydrated and autoclaved in pH 7.0 antigen retrieval solution (Histo VT One, Nacalai Tesque Inc.) at 121 °C for 10 min. The preparations were blocked with 0.3% hydrogen peroxidase in methanol for 20 min at room temperature to block endogenous peroxidase, followed by treatment with the Avidin Biotin Blocking Kit (Nichirei Bioscience Inc., Tokyo, Japan) and incubated with Blocking One (Nacalai Tesque Inc.) for 30 min to block the non-specific sites. The sections were incubated for 90 min at room temperature with either the anti-GIP_HUMAN[22–51] IgG or the control non-immune IgG purified from the preimmune serum (1:500 dilution). After washing three times in PBS, the biotinylated goat anti-rabbit IgG antibody (1:500, Vector Laboratories Inc.) was incubated for 60 min at room temperature. Antibody binding was visualised using the avidin–biotin-complex peroxidase method with the Vectastain ABC kit (Vector Laboratories Inc.) and a DAB peroxidase substrate kit (Vector Laboratories Inc.)^53^. The tissue preparations were stained with Meyer’s hematoxylin (Muto Pure Chemicals, Tokyo, Japan).

For the comparison of the exact expression sites of GIP_HUMAN[22–51] and the mature GIP using dual immunofluorescence staining, we followed the same procedures as described for normal immunohistochemical staining with tissue array, MNO341, except that we used two primary antibodies, the rabbit anti- GIP_HUMAN[22–51] IgG (1:1000) and the mouse anti-human GIP (1:500, R&D Systems) simultaneously, and two secondary antibodies, the goat anti-rabbit-Alexa Fluor 488-conjugated antibody (1:500, Abcam) and the goat anti-mouse-Alexa Fluor 594-conjugated antibody (1:1000, Abcam). The images were acquired using an LSM710 confocal microscope (Carl Zeiss).

### Statistical analysis

The data were presented as means ± SEM and were analysed using GraphPad Prism software 5.02 (GraphPad Software Inc., San Diego, CA, USA). The data between the two groups were compared using the two-tailed unpaired Student’s t-test or Welch’s t-test, and data involving three or more groups were analysed using a one-way analysis of variance followed by a Bonferroni’s post hoc test. Statistical significance was set at *P* < 0.05.

### Ethics approval and consent to participate

All the animal experimental procedures were approved by the Animal Experimentation and Ethics Committee of the Showa University School of Medicine (permission number: 09062). Procedures were performed in compliance with the ARRIVE guidelines and with guidelines for animal experiments by the Showa University School of Medicine. The study protocol using human blood samples was approved by the Ethics Committee of Kitasato University Hospital/School of Medicine (C19-245). All the study methods were undertaken in accordance with the relevant guidelines and regulations of this organisation as well as the Ethical Guidelines for Medical and Health Research Involving Human Subjects in Japan. Written informed consent was obtained from all the healthy volunteers who provided blood samples.

## Supplementary Information

Supplementary Information.
