# Supplementary material for: GIP_HUMAN[22–51] is a new proatherogenic peptide identified by native plasma peptidomics
Source: Sci Rep. 2021 Jul 14;11:14470. doi: 10.1038/s41598-021-93862-w (PMC8280211; doi:10.1038/s41598-021-93862-w)

Supplementary Information

GIP_HUMAN[22-51] is a new proatherogenic peptide identified by native plasma peptidomics

Tsuguto Masaki[1], Yoshio Kodera[2], Michishige Terasaki[3], Kazumi Fujimoto[1,2], Tsutomu Hirano[3], & Masayoshi Shichiri[1,4*]

[1]Department of Endocrinology, Diabetes and Metabolism, Kitasato University School of Medicine, 1-15-1 Kitasato, Minami-ku, Sagamihara, Kanagawa 252-0374, Japan
[2]Department of Physics, and [3]Center for Disease Proteomics, Kitasato University School of Science, 1-15-1 Kitasato, Minami-ku, Sagamihara, Kanagawa 252-0373, Japan
[3]Division of Diabetes, Metabolism and Endocrinology, Department of Medicine, Showa University School of Medicine, 1-5-8 Hatanodai, Shinagawa-ku, Tokyo 142-8555, Japan
[4]Present address, Tokyo Kyosai Hospital, 2-3-8 Nakameguro, Meguro-ku, Tokyo 153-8934, Japan

[*]Correspondence and requests for materials should be addressed to M.S. (shichiri@tkh.meguro.tokyo.jp)

Supplementary Table S1. List of 129 synthetic peptides selected *in silico* and tested for functional analyses

No.	Peptide sequence	Uniprot entry name(s) [Position]	Gene name	Protein Name
1	MEPLGRQLTSGP	A2AP_HUMAN[0028-0039]	SERPINF2	Alpha-2-antiplasmin
2	EPLGRQLTSGP	A2AP_HUMAN[0029-0039]	SERPINF2	Alpha-2-antiplasmin
3	LGRQLTSGP	A2AP_HUMAN[0031-0039]	SERPINF2	Alpha-2-antiplasmin
4	MSLSSFSVNRPFLFF	A2AP_HUMAN[0404-0418]	SERPINF2	Alpha-2-antiplasmin
5	AGAFCLSEDAGLGISSTASLR	A2MG_HUMAN [767-787];<br>PZP_HUMAN [773-793]	A2M; PZP	Alpha-2-macroglobulin; Pregnancy zone protein
6	SVSGKPQYMV	A2MG_HUMAN[0024-0033]	A2M	Alpha-2-macroglobulin
7	YESDVMGR	A2MG_HUMAN[0708-0715]	A2M	Alpha-2-macroglobulin
8	TEVPSVPEHGRKDTVIKPLL	A2MG_HUMAN[0885-0904]	A2M	Alpha-2-macroglobulin
9	VPEHGRKDTVIKPLLVEPEG	A2MG_HUMAN[0890-0909]	A2M	Alpha-2-macroglobulin
10	AHTSFQISLSVSYTGSR	A2MG_HUMAN[1357-1373]	A2M	Alpha-2-macroglobulin
11	GTHVDLGLASANVDFAFSLYK	AACT_HUMAN[0042-0062]	SERPINA3	Alpha-1-antichymotrypsin
12	LVETRTIVRF	AACT_HUMAN[0386-0395]	SERPINA3	Alpha-1-antichymotrypsin
13	VETRTIVRFNRPFLMIIVPTDTQNIFFMS	AACT_HUMAN[0387-0415]	SERPINA3	Alpha-1-antichymotrypsin
14	RTIVRFNRPFLMIIVPTDTQNIFFMSKVTNPKQA	AACT_HUMAN[0390-0423]	SERPINA3	Alpha-1-antichymotrypsin
15	RFNRPFLMIIVPTDTQNIFFMSKV	AACT_HUMAN[0394-0417]	SERPINA3	Alpha-1-antichymotrypsin
16	NRPFLMIIVPTDTQNIFFMSKVTNPKQA	AACT_HUMAN[0396-0423]	SERPINA3	Alpha-1-antichymotrypsin
17	IIVPTDTQNIFFMSKVTNPKQA	AACT_HUMAN[0402-0423]	SERPINA3	Alpha-1-antichymotrypsin
18	DAHKSEVAHRF-NH2	ALBU_HUMAN[0025-0035]	ALB	Serum albumin
19	DAHKSEVAHRFKDL-NH2	ALBU_HUMAN[0025-0038]	ALB	Serum albumin
20	DAHKSEVAHRFKDLGEEN-NH2	ALBU_HUMAN[0025-0042]	ALB	Serum albumin
21	FAEEGKKLVAASQAALGL	ALBU_HUMAN[0592-0609]	ALB	Serum albumin
22	KPEVLEVTLNRPFLF	ANGT_HUMAN[0448-0462]	AGT	Angiotensinogen
23	KPEVLEVTLNRPFLFAVYDQSATALHFLGRVANPLSTA	ANGT_HUMAN[0448-0485]	AGT	Angiotensinogen
24	AVYDQSATALHFLGRVANPLSTA	ANGT_HUMAN[0463-0485]	AGT	Angiotensinogen
25	AFLEVNEEGSEAAASTAVVIAGR	ANT3_HUMAN[0403-0425]	SERPINC1	Antithrombin-III
26	HSSLAFWKTDASDVKPC	APOH_HUMAN[0329-0345]	APOH	Beta-2-glycoprotein 1
27	SLAFWKTDASDVKPC	APOH_HUMAN[0331-0345]	APOH	Beta-2-glycoprotein 1
28	FWKTDASDVKPC	APOH_HUMAN[0334-0345]	APOH	Beta-2-glycoprotein 1
29	SHTLRTTCWDGKLEYPTCA	CFAH_HUMAN [1211-1229];<br>FHR1_HUMAN [310-328]	CFH; CFHR1	Complement factor H; Complement factor H-related protein 1
30	RPHFFFPKSRIV	CLUS_HUMAN[0215-0226]	CLU	Clusterin
31	PITVTVPVEVSRKNPKFMETVAEKALQEYRKKHREE	CLUS_HUMAN[0414-0449]	CLU	Clusterin
32	EDVSAGEDCGPLPEGGPEPRSDGAKPGPRE-NH2	COLI_HUMAN [0105-0134]	POMC	Pro-opiomelanocortin
33	YDPEAASAPGSGNPCHEASAAQKENAGEDPGLAR	DCD_HUMAN[0020-0053]	DCD	Dermcidin
34	SLLEKGLDGAKKAV	DCD_HUMAN[0064-0077]	DCD	Dermcidin
35	LEKGLDGAKK	DCD_HUMAN[0066-0075]	DCD	Dermcidin
36	ESVGKGAVHDVKD	DCD_HUMAN[0092-0104]	DCD	Dermcidin
37	KPGVYTDVAYYLAWIREHTVS	FA12_HUMAN[0595-0615]	F12	Coagulation factor XII
38	DHENANKILNRPK	FA9_HUMAN[0033-0045]	F9	Coagulation factor IX
39	ADANLEAGNVKETRAS	FBN1_HUMAN[0025-0040]	FBN1	Fibrillin-1
40	NLEAGNVKE	FBN1_HUMAN[0028-0036]	FBN1	Fibrillin-1
41	NLEAGNVKETRASRA	FBN1_HUMAN[0028-0042]	FBN1	Fibrillin-1
42	TNTNVNCPIECFMPLDVQADREDSRE	FINC_HUMAN[2452-2477]	FN1	Fibronectin
43	TNVNCPIECFMPLDVQA	FINC_HUMAN[2454-2470]	FN1	Fibronectin
44	TNVNCPIECFMPLDVQADREDSRE	FINC_HUMAN[2454-2477]	FN1	Fibronectin
45	ATASRGASQAGAPQGR	GELS_HUMAN[0028-0043]	GSN	Gelsolin
46	PLDRAMAELA	GELS_HUMAN[0772-0781]	GSN	Gelsolin
47	EKKEGHFSALPSLPVGSHAKVSSPQPR	GIP_HUMAN[0022-0048]	GIP	Gastric inhibitory polypeptide
48	EKKEGHFSALPSLPVGSHAKVSSPQPRGPR	GIP_HUMAN[0022-0051]	GIP	Gastric inhibitory polypeptide
49	ALPSLPVGSHAKVSSPQPRGPR	GIP_HUMAN[0030-0051]	GIP	Gastric inhibitory polypeptide
50	SLPVGSHAKVSSPQPRGPR	GIP_HUMAN[0033-0051]	GIP	Gastric inhibitory polypeptide
51	SSPAKNPSDEDLLR	GIP_HUMAN[0117-0130]	GIP	Gastric inhibitory polypeptide
52	LPPTSAHGNVAEGETKPD	HEMO_HUMAN[0026-0043]	HPX	Hemopexin
53	PPTSAHGNVAEGETKPD	HEMO_HUMAN[0027-0043]	HPX	Hemopexin
54	FPSPVDAAFRQGHN	HEMO_HUMAN[0093-0106]	HPX	Hemopexin
55	SVFLIKGDKVWVYPPEK	HEMO_HUMAN[0107-0123]	HPX	Hemopexin
56	SVFPQQTGQLAELQPQDRAGARASWMPMFQ	HEPC_HUMAN[0025-0054]	HAMP	Hepcidin
57	KAVGDKLPECEAVCGKPKNPANPVQ	HPT_HUMAN [136-160];<br>HPTR_HUMAN [78-102]	HP; HPR	Haptoglobin; Haptoglobin-related protein
58	GKPKNPANPVQ	HPT_HUMAN [150-160];<br>HPTR_HUMAN [92-102]	HP; HPR	Haptoglobin; Haptoglobin-related protein
59	GKPKNPANPVQR	HPT_HUMAN [150-161];<br>HPTR_HUMAN [92-103]	HP; HPR	Haptoglobin; Haptoglobin-related protein
60	WVQKTIAEN	HPT_HUMAN [398-406];<br>HPTR_HUMAN [340-348]	HP; HPR	Haptoglobin; Haptoglobin-related protein
61	AVIALLLWGQ	HPT_HUMAN[0006-0015]	HP	Haptoglobin
62	VDSGNDVTDIAD	HPT_HUMAN[0019-0030]	HP	Haptoglobin
63	VDSGNDVTDIADD	HPT_HUMAN[0019-0031]	HP	Haptoglobin
64	VDSGNDVTDIADDG	HPT_HUMAN[0019-0032]	HP	Haptoglobin
65	VDSGNDVTDIADDGCPKPP	HPT_HUMAN[0019-0037]	HP	Haptoglobin

66	VDSGNDVTDIADDGCPKPPEIAHG	HPT_HUMAN[0019-0042]	HP	Haptoglobin
67	NEKQWINKAVGDKLPECEAVCGKPKNPANPVQ	HPT_HUMAN[0129-0160]	HP	Haptoglobin
68	SIQDWVQKTIAEN	HPT_HUMAN[0394-0406]	HP	Haptoglobin
69	FTHTFPK	HRG_HUMAN[0519-0525]	HRG	Histidine-rich glycoprotein
70	FFQYDTWKQSTQ	IGF2_HUMAN[0113-0124]	IGF2	Insulin-like growth factor II
71	FFQYDTWKQSTQRL	IGF2_HUMAN[0113-0126]	IGF2	Insulin-like growth factor II
72	YDTWKQSTQRL	IGF2_HUMAN[0116-0126]	IGF2	Insulin-like growth factor II
73	DTWKQSTQRL	IGF2_HUMAN[0117-0126]	IGF2	Insulin-like growth factor II
74	FVNQHLCGS	INS_HUMAN[0025-0033]	INS	Insulin
75	FVNQHLCGSHLVEA	INS_HUMAN[0025-0038]	INS	Insulin
76	LVCGERGFFYTPKT	INS_HUMAN[0041-0054]	INS	Insulin
77	EAEDLQVGQVELGGGPGAGSLQP	INS_HUMAN[0057-0079]	INS	Insulin
78	EAEDLQVGQVELGGGPGAGSLQPLALEGSLQ	INS_HUMAN[0057-0087]	INS	Insulin
79	DLQVGQVELGGGPGAGSLQPLALEGSLQ	INS_HUMAN[0060-0087]	INS	Insulin
80	GAGSLQPLALEGSLQ	INS_HUMAN[0073-0087]	INS	Insulin
81	EQCCTSICSL	INS_HUMAN[0093-0102]	INS	Insulin
82	QCCTSICSL	INS_HUMAN[0094-0102]	INS	Insulin
83	SARLNSQRLVFNRPFLMFIVDNNILFLGKVNRP	IPSP_HUMAN[0374-0406]	SERPINA5	Plasma serine protease inhibitor
84	SQRLVFNRPFLMFIVDNNILFLGKVNRP	IPSP_HUMAN[0379-0406]	SERPINA5	Plasma serine protease inhibitor
85	TAFISDFAVTADGNAFIGDIK	ITIH1_HUMAN[0094-0114]	ITIH1	Inter-alpha-trypsin inhibitor heavy chain H1
86	FEIPINGLSEFVDYEDLVELAPGKFQLVAENRRYQ	ITIH2_HUMAN[0019-0053]	ITIH2	Inter-alpha-trypsin inhibitor heavy chain H2
87	LGFSEVELVQMVVDGVK	KCRB_HUMAN[0342-0358]	CKB	Creatine kinase B-type
88	YRITEATKTVG	KNG1_HUMAN[0057-0067]	KNG1	Kininogen-1
89	MISLMKRPPGFSPFR	KNG1_HUMAN[0375-0389]	KNG1	Kininogen-1
90	MKRPPGFSPFRS	KNG1_HUMAN[0379-0390]	KNG1	Kininogen-1
91	MKRPPGFSPFRSSRIG	KNG1_HUMAN[0379-0394]	KNG1	Kininogen-1
92	RPPGFSPFR	KNG1_HUMAN[0381-0389]	KNG1	Kininogen-1
93	RPPGFSPFRSS	KNG1_HUMAN[0381-0391]	KNG1	Kininogen-1
94	RPPGFSPFRSSR	KNG1_HUMAN[0381-0392]	KNG1	Kininogen-1
95	DDDWIPDIQID	KNG1_HUMAN[0584-0594]	KNG1	Kininogen-1
96	DFPDTTSPK-NH2	KNG1_HUMAN[0605-0613]	KNG1	Kininogen-1
97	VGGTGGIGGVGGTGGVGNRAPR	MMRN1_HUMAN[0159-0180]	MMRN1	Multimerin-1
98	GGVGGTGGVGNRAPR	MMRN1_HUMAN[0166-0180]	MMRN1	Multimerin-1
99	LTFPLDYHLNQPFIFVLRDTDTGALLFIGKILDPRGP	PEDF_HUMAN[0382-0418]	SERPINF1	Pigment epithelium-derived factor
100	EPPPRTLPATDLQ	PGRP2_HUMAN[0564-0576]	PGLYRP2	N-acetylmuramoyl-L-alanine amidase
101	GQGIHHAAGQVGK	SBSN_HUMAN[0225-0237]	SBSN	Suprabasin
102	GQGAHHAAGQAGNEAGR	SBSN_HUMAN[0243-0259]	SBSN	Suprabasin
103	GQGVHHTAGQVGKEAE	SBSN_HUMAN[0279-0294]	SBSN	Suprabasin
104	GQGVHHTAGQVGKEAEK	SBSN_HUMAN[0279-0295]	SBSN	Suprabasin
105	GQGVHHTAGQVGKEAEKF	SBSN_HUMAN[0279-0296]	SBSN	Suprabasin
106	GHDIHHTAGQAGKEGD	SBSN_HUMAN[0423-0438]	SBSN	Suprabasin
107	KLGQGAHHAAGQAG	SBSN_HUMAN[0511-0524]	SBSN	Suprabasin
108	KLGQGAHHAAGQAGK	SBSN_HUMAN[0511-0525]	SBSN	Suprabasin
109	SNPPKPKGKPPAPKPASPKKNIKT	SFRP4_HUMAN[0311-0334]	SFRP4	Secreted frizzled-related protein 4
110	STSSLLEACTFRRP	TRFE_HUMAN[0685-0698]	TF	Serotransferrin
111	TSSLLEACTFRRP	TRFE_HUMAN[0686-0698]	TF	Serotransferrin
112	SSLLEACTFRRP	TRFE_HUMAN[0687-0698]	TF	Serotransferrin
113	FTANDSGPRRYTIAALLSPYSYSTT	TTHY_HUMAN[0115-0139]	TTR	Transthyretin
114	NDSGPRRYTIAALLSPYSYSTTAVVTNPKE	TTHY_HUMAN[0118-0147]	TTR	Transthyretin
115	GPRRYTIAALLSPYSYST	TTHY_HUMAN[0121-0138]	TTR	Transthyretin
116	AALLSPYSYSTTAVVTNPKE	TTHY_HUMAN[0128-0147]	TTR	Transthyretin
117	SPYSYSTTAVVTNPKE	TTHY_HUMAN[0132-0147]	TTR	Transthyretin
118	SYSTTAVVTNPKE	TTHY_HUMAN[0135-0147]	TTR	Transthyretin
119	TAVVTNPKE	TTHY_HUMAN[0139-0147]	TTR	Transthyretin
120	AVVTNPKE	TTHY_HUMAN[0140-0147]	TTR	Transthyretin
121	MRCGGCCNDEGLECVPT	VEGFA_HUMAN[0081-0097]	VEGFA	Vascular endothelial growth factor A
122	GRPEAQPPPLSSEHKEPVAGDAVPGPKDGSAPEVRGA	VGF_HUMAN[0026-0062]	VGF	Neurosecretory protein VGF
123	GGEERVGEEDEEAAEAEAEAEEAERARQNA	VGF_HUMAN[0373-0402]	VGF	Neurosecretory protein VGF
124	NAPPEPVPPPRAAPAPTHV	VGF_HUMAN[0485-0503]	VGF	Neurosecretory protein VGF
125	LLQDSVDFSLADAINTEFK	VIME_HUMAN[0079-0097]	VIM	Vimentin
126	SDFASNCCSINSPPLYCDSEIDAELKNIL	VTDB_HUMAN[0446-0474]	GC	Vitamin D-binding protein
127	INSPPLYCDSEIDAELKNIL	VTDB_HUMAN[0455-0474]	GC	Vitamin D-binding protein
128	SPPLYCDSEIDAELKNIL	VTDB_HUMAN[0457-0474]	GC	Vitamin D-binding protein
129	PPYPRSIAQYWLGCPAPGHL	VTNC_HUMAN[0459-0478]	VTN	Vitronectin

Supplementary Table S2. Baseline laboratory data of *ApoE*$^{-/-}$ mice infused with or without GIP_HUMAN[22–51] and/or anti- GIP_HUMAN[22–51].

	Vehicle	GIP_HUMAN[22–51]	GIP_HUMAN[22–51]+ anti-GIP_HUMAN[22–51] IgG	anti-GIP_HUMAN[22–51] IgG
HDL-cholesterol (mg/dL)	23.9 ± 5.7	22.0 ± 5.4	28.3 ± 3.8	44.8 ± 9.8
Total cholesterol (mg/dL)	1510 ± 193	1394 ± 118	1355 ± 182	1576 ± 61
Triglyceride (mg/dL)	329 ± 87.9	311 ± 60.5	260 ± 65.7	191 ± 47.6
Insulin (ng/mL)	0.34 ± 0.009	0.35 ± 0.015	0.37 ± 0.026	0.33 ± 0.009
Total-GIP (pg/mL)	223 ± 24.3	209 ± 28.2	357 ± 45.4 *	263 ± 25.4
Glucose (mg/dL)	87.0 ± 5.7	84.0 ± 6.7	91.0 ± 4.3	86.8 ± 13.2

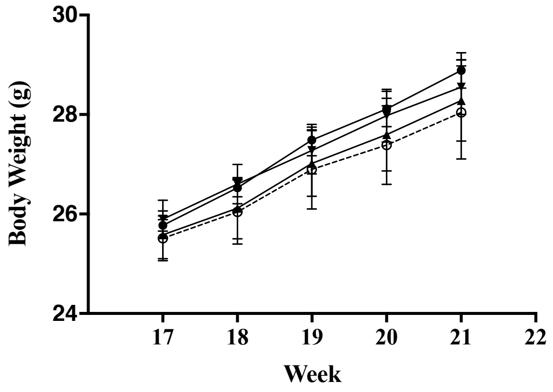

Supplementary Figure 1. Body weight changes for the four groups of *ApoE*$^{-/-}$ mice infused with or without GIP_HUMAN[22–51] and or anti-GIP_HUMAN[22–51]. The 17-week-old *ApoE*$^{-/-}$ mice were infused with saline alone (open circles), GIP_HUMAN[22–51] (closed circles), GIP_HUMAN[22–51] plus anti-GIP_HUMAN[22–51] IgG (closed triangles) or anti-GIP_HUMAN[22–51] IgG (closed inverted triangles) by osmotic mini-pumps for four weeks and their body weights measured before and every week. Data represent mean ± S.E.M.

Uncropped Figures

Uncropped photographs of the protein blots shown in Figure 2e and 2f.

Figure 2e

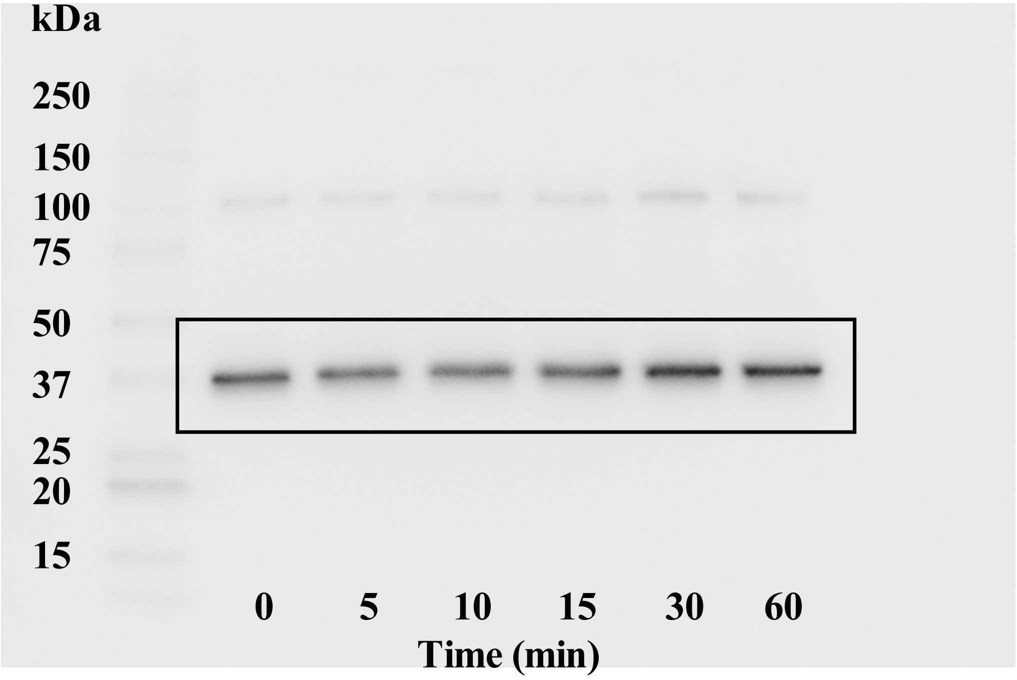

Figure 2f

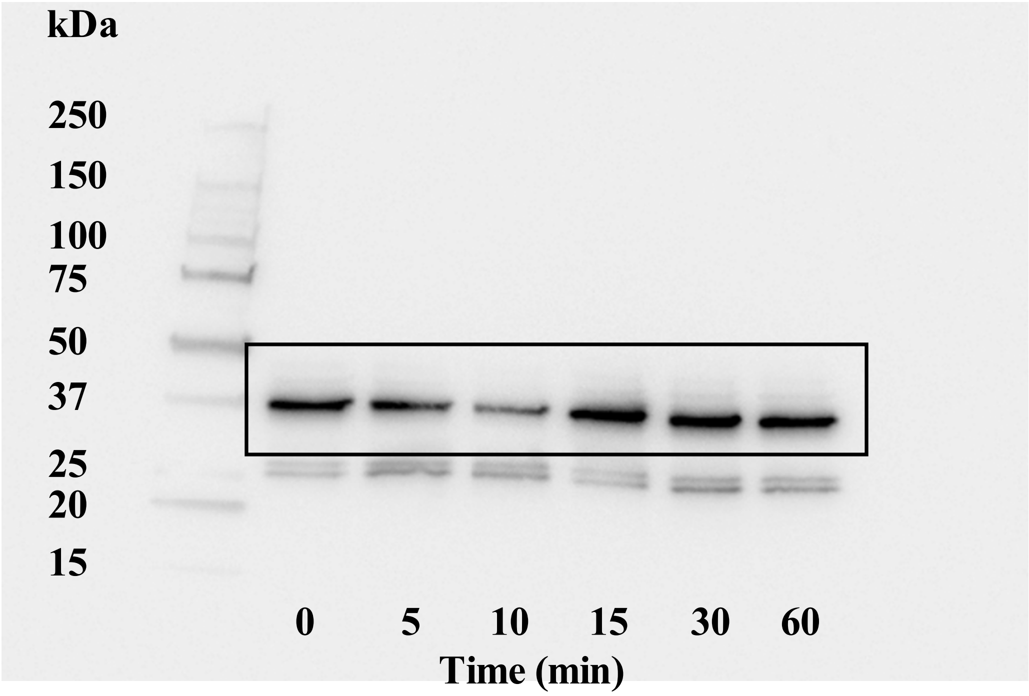

Supplement: Supplementary file 1 — Supplementary Information. [file 41598_2021_93862_MOESM1_ESM.pdf]
